# Isolation and full-genome sequencing of Seneca Valley virus in piglets from China, 2016

**DOI:** 10.1186/s12985-016-0631-2

**Published:** 2016-10-19

**Authors:** Suhong Qian, Wenchun Fan, Ping Qian, Huanchun Chen, Xiangmin Li

**Affiliations:** 1 Division of Animal Virology, College of Veterinary Medicine, Huazhong Agricultural University, Wuhan, Hubei People’s Republic of China; 2 State Key Laboratory of Agricultural Microbiology, Huazhong Agricultural University, Wuhan, Hubei People’s Republic of China; 3 The Cooperative Innovation Center for Sustainable Pig Production, Wuhan, Hubei People’s Republic of China

**Keywords:** Emerging disease, Vesicular disease, Seneca valley virus, Swine

## Abstract

**Background:**

Seneca valley virus (SVV), a member of the *Picornaviridae* family, is a small non-enveloped RNA virus, that is linked to porcine idiopathic vesicular disease (PIVD). SVV infection in swine results in vesicular disease and epidemic transient neonatal losses (ETNL). The first case of SVV infection was reported in Guangdong, South China in 2015.

**Results:**

We isolated and characterized an SVV HB-CH-2016 strain from vesicular lesion tissue specimens from piglets with PIVD in Hubei, Central China. The complete genome sequence of SVV HB-CH-2016 strain shares high nucleotide identities (94 to 99 %) with all previously reported SVV genomes, moreover, the polyprotein accounts for 98–99 % of amino acid sequence identity. Therefore, the SVV HB-CH-2016 strain is closely related to the SVV CH-01-2015 strain.

**Conclusions:**

The case reported in this paper is the second SVV infection case in China. Our findings demonstrate that sporadic SVV infection has occurred in Central China, and therefore, active surveillance on the swine population is important. Moreover, veterinarians must pay attention to this vesicular disease and reinforce biosecurity measures and prevent SVV spread.

**Electronic supplementary material:**

The online version of this article (doi:10.1186/s12985-016-0631-2) contains supplementary material, which is available to authorized users.

## Background

Seneca valley virus (SVV), a small, non-enveloped virus with a single-stranded and positive-sense RNA that 7.2 kb long, belongs to the *Senecavirus* genus of the family *Picornavirus* [1]. Its genome forms a single, long open-reading frame (ORF) flanked by 5′-and 3′-untranslated regions (UTRs). The translation of the SVV genome by host cellular machinery produces a polyprotein with 2181 amino acids (aa). The polyprotein is cleaved by viral and cellular proteases to produce 12 mature proteins in an order from 5′ to 3′ is L protein (L)-1A-1B-1C-1D-2A-2B-2C-3A-3B-3C-3D [1]. SVV has been linked to PIVD outbreaks of in many countries, including Canada, the Unites States, Australia, New Zealand, Italy and Brazil [2–9]. In the summer of 2015, the first case of SVV infection was reported in swine farms in Guangdong, South China [9]. In this study, we isolated and characterized SVV from a piglet with an idiopathic vesicular disease but not infected with vesicular stomatitis virus (VSV), foot-and-mouth disease virus (FMDV) and swine vesicular disease virus (SVDV).

## Methods

In March 2016, an outbreak of vesicular disease occurred among piglets in a swine farm in Hubei province. To determine etiology, vesicular lesion swab specimens were collected and detected using VSV, FMDV and SVDV specific primers. Total RNA from the specimens was isolated using the TRIzol reagent (Invitrogen, Grand Island, NY, USA) and 1.0 μg of total RNA was reverse-transcribed using a First Strand cDNA Synthesis Kit (TOYOBO, Japan) according to the manufacturer’s instructions. PCR amplification was performed using various virus-specific primers (listed in Additional file 1: Table S1). Virus isolation was carried out in a BHK-21 cell culture system from the SVV-positive specimens and labeled HB-CH-2016. Mouse anti-SVV VP1 polyclonal antibody was prepared in our lab using recombinant SVV VP1 protein. Identification of SVV BH-CH-2016 using immunofluorescence assay (IFA), Western blot analysis, and plaque assay was carried out as described previously [10]. Complete genome-sequencing was performed using 8 pairs of overlapping primers (Additional file 1: Table S1) based on the SVV CH-01-2015 strain (GenBank accession number:KT321458). The phylogenetic tree was constructed by the neighbor-joining method, with 1000 bootstrap replicates, using MEGA6.0 software.

## Results

**Fig. 1:**
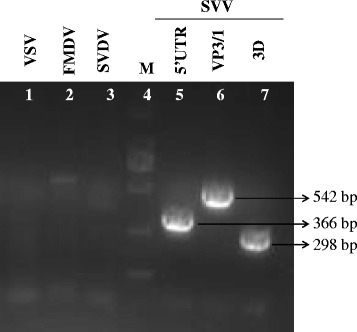
PCR detection of Seneca valley virus from swine vesicular lesion tissue samples. PCR amplification identified the causative agent in the swine vesicular lesion tissues using VSV, FMDV, SVDV and SVV specific primers. Lane 1 is for VSV (638 bp). Lane 2 is for FMDV (422 bp). Lane 3 is for SVDV (861 bp). Lane 4 is DNA marker (from top to bottom is 2000-1000-750-500-250-100 bp). Lanes 5 to 7 are for SVV specific 5′ UTR (366 bp), VP3/1 (542 bp), and 3D (298 bp) gene, respectively

**Fig. 2:**
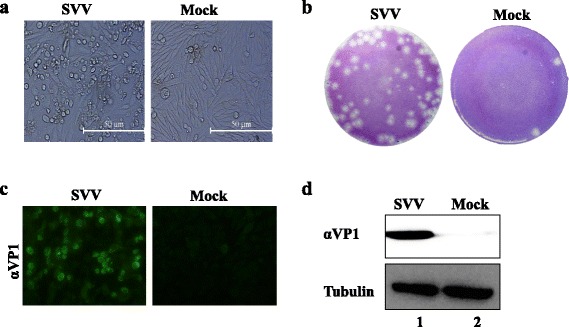
Identification of SVV HB-CH-2016 strain. **a** The cytopathic effect of BHK-21 cells infected with SVV HB-CH-2016 strain at 18 h post-infection. **b** Plaque morphology in BHK-21 cells infected with fourth-passage SVV HB-CH-2016 strain at 48 h post-infection. **c** Immunofluorescence assay (IFA) of BHK-21 cells infected with SVV HB-CH-2016 strain at 12 h post-infection. Cells were stained with primary antibody using home-made mouse anti-SVV VP1 polyclonal antibodies. **d** Western blot analysis of BHK-21 cells infected with SVV HB-CH-2016 strain at 12 h post-infection. Cells were stained with primary antibody using a home-made mouse polyclonal anti-SVV VP1 antibody and mouse anti-tubulin antibody

We successfully isolated the infectious SVV HB-CH-2016 strain from piglets with PIVD, which was negative for VSV, FMDV or SVDV, but positive for SVV (Fig. 1). As shown in Fig. 2a, the fourth passage SVV isolate induced typical cytopathic effects characterized by rounding and shrinkage and degeneration of BHK-21 cells at 18 h post-infection. The viral titer of fourth passage SVV HB-CH-2016 strain was 5 × 10^7^ PFU/mL. The plaque morphology was distinct and plaque size was approximately 1.0–2.0 mm in BHK-21 cells (Fig. 2b). To confirm the isolation of SVV, immunofluorescence assay and Western blot analysis were conducted using home-made mouse polyclonal anti-SVV VP1 antibody. As shown in Fig. 2c, cells infected by the isolate reacted with the specific mouse polyclonal antibody against SVV VP1 protein by IFA. Meanwhile, Western blot analysis showed approximately 30 kilodalton (kDa) band in cells infected with the isolate (Fig. 2d, lane 1) but not in the mock cells (Fig. 2d, lane 2). These results indicated successful isolation of the infectious SVV HB-CH-2016 strain.

**Fig. 3:**
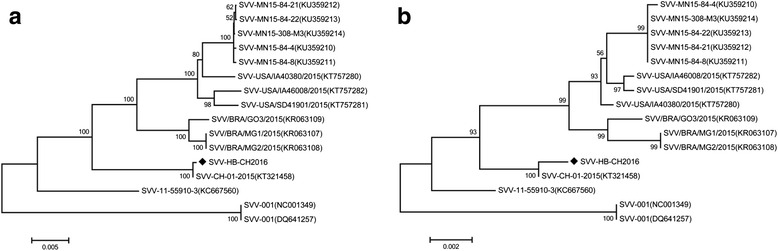
Phylogenetic analysis of SVV complete nucleotides and amino acid sequences. Phylogenetic trees were constructed using the neighbor-joining method, with 1000 bootstrap replicates, using MEGA6.0 software. The newly isolated SVV HB-CH-2016 strain is marked ◆. The number on every 100 branches indicates bootstrap values. **a** Phylogenetic analyses of SVV based on full-genome nucleotides. **b** Phylogenetic analyses of SVV based on full-polyprotein amino acids. Reference sequences retrieved from the GenBank are indicated by years of isolation, origins and accession numbers

The complete genome of SVV HB-CH-2016 strain has been amplified, sequenced and deposited in GenBank (accession number: KX377924). The strain consists of 7300 nucleotides (nt) in length, including a 668-nt 5′ UTR, an opern reading frame (ORF) encoding a 2181 amino acid polyprotein and a 86-nt 3′ UTR cantains a partial poly(A) sequence. Analysis of the full-genome sequence of SVV HB-CH-2016 strain indicated that it shares high nucleotide identities (94 to 99 %) with all previous SVV genomes in the GenBank (Fig. 3a), and the polyprotein shares 98–99 % amino acid sequence identity (Fig. 3b). Two phylogenetic tree results were constructed based on a complete genome sequence or ployprotein amino acid sequence and demonstrated that the SVV HB-CH-2016 strain is closely related to SVV CH-01-2015.

## Discussion

SVV infection in piglets cuases neonatal losses and the mortality rate of 1 ~ 4-day-old pigs is approximately 30 ~ 70 % [8, 11]. Currently, swine vesicular disease outbreaks are linked to SVV, and swine SVV infection has been reported in Canada, the United States, Brazil, Australia, New Zealand and Italy. Moreover, the first case of SVV infection has been reported in South China in 2015 [9], and sporadichave been observed in Hubei province in March 2016, Central China. Therefore, active surveillance for SVV in swine populations is important and veterinarians must be alert to this vesicular disease in China. As an emerging virus, SVV has been poorly understood, specifically with regard to its transmission, pathogenesis and immunobiology. In response, investigating the relationship between evolutionary dynamics, pathogenesis and epidemiological features of SVV infection, is crucial in facilitating the development of antiviral strategies and offering effective control measures against SVV infection.

## Additional file

Additional file 1: Table S1. List of primers used in the study. (DOCX 19 kb)
