# Supplementary material for: Isolation and full-genome sequencing of Seneca Valley virus in piglets from China, 2016
Source: Virol J. 2016 Oct 19;13:173. doi: 10.1186/s12985-016-0631-2 (PMC5069920; doi:10.1186/s12985-016-0631-2)
Supplement: Additional file 1: Table S1. — List of primers used in the study. (DOCX 19 kb) [file 12985_2016_631_MOESM1_ESM.docx]

**Table S1:** List of primers used in the study

| Primer Name | Primer Sequence (5′to 3′) |
| --- | --- |
| VSV-F^a^ | GTCAAGAGAATCATTGAC |
| VSV-R^a^ | GTTCCGTATCTGAACGA |
| FMDV-F^b^ | ATGCAGGAGGACATGTCAAC |
| FMDV-R^b^ | TTGATGTCACGTGCTTTGAG |
| SVDV-F^c^ | TTCTTCCAAGGGCCCCCAGG |
| SVDV-R^c^ | GCCTGTGGTTTTCATGGTTGT |
| 5′UTR-F^d^ | GTTGTAACTACAAGATTTAGC |
| 5′UTR-R^d^ | CAGGCAGTATCCAAGGCACG |
| VP3/1-F^e^ | TTCCACTCCACCGACAACG |
| VP3/1-R^e^ | GATACCTTCCCACCCTTGC |
| 3D-F^f^ | CAAGGAATTTGAATATGACATGG |
| 3D-R^f^ | GCAGCTTCTCGAGTAGTGTTCC |
| SVV-1F^g^ | TTTGAAATGGGGGGCTGGGCCCTC |
| SVV-1R^g^ | GTCGAGAAGTCTACTGAATTCTG |
| SVV-2F^h^ | AGGGTAATGTTCAGACAACCTC |
| SVV-2R^h^ | GGGGGACAAGCACCATAACGAGG |
| SVV-3F^i^ | CACACCAAATTCTGAACGCGAG |
| SVV-3R^i^ | AGAAATCGGTGTCAGTGTTACC |
| SVV-4F^j^ | CTCTGCTGGTGAGGATTACACC |
| SVV-4R^j^ | AGTCTGTGTAATTGAGCCCTCG |
| SVV-5F^k^ | CAGACCTGGAGAAAGTATGTG |
| SVV-5R^k^ | TGGGCAGCAAAAGGATAAATGG |
| SVV-6F^l^ | CTGATCCTTCTGCGGTCTCTCG |
| SVV-6R^l^ | GCATTGTAGCCAGAGGTTCAC |
| SVV-7F^m^ | ACAAGGGATGGTGCGGTTCGG |
| SVV-7R^m^ | GATCACATTGTTGAGCACTGTG |
| SVV-8F^n^ | CTCATCTCTCACTTTTTCACC |
| SVV-8R^n^ | TTTTTTTCCCTTTTCCCTTTTCTGTTCCGACTG |

a. Primers used to the detect VSV in the tissue samples; b. Primers used to the detect FMDV in the tissue samples; C. Primers used to the detect SVDV in the tissue samples; d~f. Primers used to the detect SVV in the tissue samples; g~n. 8 pairs of primer used to clone the complete genome of SVV HB-WH-2016 stain.

F: forward, R: reverse.
